# De Novo *ACTB* Variant Associated With Juvenile-Onset Temporal Lobe Epilepsy With Favorable Outcomes

**DOI:** 10.1155/humu/9951922

**Published:** 2025-02-12

**Authors:** Hong-Jun Yan, Peng-Yu Wang, Wen-Hui Liu, Yu-Jie Gu, Jia-Cheng Pan, Hua Li, Sheng Luo

**Affiliations:** ^1^Epilepsy Center, Guangdong Sanjiu Brain Hospital, Guangzhou, Guangdong, China; ^2^Institute of Neuroscience, Key Laboratory of Neurogenetics and Channelopathies of Guangdong Province and the Ministry of Education of China, The Second Affiliated Hospital, Guangzhou Medical University, Guangzhou, Guangdong, China; ^3^School of Computer and Information Engineering, Nanjing University of Technology, Nanjing, China

**Keywords:** *ACTB*, correlation between spatiotemporal expression and phenotypes, hippocampal sclerosis, phenotypic spectrum, temporal lobe epilepsy

## Abstract

Genetic factors are estimated to contribute to 80% of people with epilepsy. However, only four genes were reported to be associated with temporal lobe epilepsy (TLE). This study is aimed at investigating the association between *ACTB* and TLE. Trio-based exome sequencing was performed in a patient, and a de novo *ACTB* variant was identified. The patient presented with TLE featuring by age of onset in juvenile, seizure-free status in adulthood, complications of memory decline and irritability, epileptic discharges in the bilateral temporal lobes, and bilateral hippocampal sclerosis. The pathogenicity of the identified *ACTB* variant was supposed by multiple pieces of evidence, including the missense tolerance ratio of 0%, high conservation of the affected residue, predicted to be “damaging” or “conserved” by 17 in silico tools, and classification of likely pathogenic variant by the American College of Medical Genetics and Genomics (ACMG) guidelines. Protein modeling indicated the alteration of protein structure and stability caused by the identified variant. The spatiotemporal expression of *ACTB* is consistent with the phenotypic features of this patient. This study suggested that *ACTB* is a novel candidate causative gene of TLE. The correlation between phenotypes and spatial–temporal expression provides a novel perspective for further exploration of the pathogenesis and prognosis of the disease.

## 1. Introduction

Temporal lobe epilepsy (TLE) is a type of seizure disorder, characterized by recurrent epileptic activity originating from the temporal lobe of the brain. TLE is a common focal epilepsy, affecting a significant proportion of the population, particularly in adulthood and juveniles [1]. Seizures of TLE are often intractable and could cause cognitive, emotional, and behavioral impairments, which significantly impact patient's quality of life [1, 2]. The etiology of TLE is complex, encompassing factors such as trauma and infection. However, in recent years, genetic factors have emerged as a significant contributor to the pathogenesis of TLE [3]. Genetic studies have reported several genes possibly associated with the occurrence of TLE, such as *CPA6* (Mendelian Inheritance in Man (MIM): 609562), *GAL* (MIM: 137035), *LGI1* (MIM: 604619), and *RELN* (MIM: 600514). While these genetic discoveries have significantly advanced our understanding of TLE, it is important to note that they can only explain a small portion of cases [4]. The etiology of TLE remains incompletely understood, and further research is needed to identify additional genetic factors. Such insights are expected to lead to the development of more targeted and effective treatment strategies for patients with TLE.

The *ACTB* gene, encoding the beta-actin protein, holds a pivotal position in cellular functions. It plays a vital role in facilitating cell motility, maintaining cell shape, and regulating intracellular signaling [5]. Beta-actin, as a cytoskeletal protein, is integral to the structural stability and functional versatility of cells, particularly neurons, which rely on its presence for proper functioning [6]. This gene is expressed ubiquitously, including in the brain. Notably, studies in mice have demonstrated that homozygous null mutants of the *Actb* gene result in embryonic lethality with complete penetrance [7], emphasizing its indispensable role.

In humans, increasing evidence suggests links between *ACTB* variants and human diseases. Variants of *ACTB* have been associated with three neurological disorders, including Baraitser–Winter syndrome 1 (BRWS1 (MIM: 243310)), dystonia-deafness syndrome 1 (DDS1 (MIM: 607371)), and thrombocytopenia 8 (THC8), with dysmorphic features and developmental delay (MIM: 620475). Baraitser–Winter syndrome (BRWS) is a rare developmental syndrome, featuring distinct physical features and variable muscular, intellectual, and epileptic issues [8]. DDS1 is characterized by juvenile-onset sensorineural deafness with later onset of progressive dystonia that often involves the bulbar region, resulting in dysarthria and dysphagia [9]. THC8 is a syndromic disorder characterized by early childhood onset of thrombocytopenia, enlarged platelets, dysmorphic facial features, and developmental and speech delays [10]. Seizures were occasionally exhibited in patients with the three severe disorders. Generally, the phenotypic spectrum of genes is broadly distributed, encompassing both severe and mild phenotypes. However, it is unknown whether *ACTB* is associated with mild phenotypes, such as TLE.

In this study, trio-based whole-exome sequencing (WES) was performed in a patient with TLE but without acquired causes. One novel *de novo* likely pathogenic variant in the *ACTB* gene was identified in a TLE case, with features including the age of onset in juvenile and seizure-free status in adulthood. The pathogenicity of the identified *ACTB* variant was assessed from multiple aspects, including the missense tolerance ratio, the conservation of the affected residue, hydrophobicity alteration, prediction of 18 in silico tools, and classification of the American College of Medical Genetics and Genomics (ACMG) guidelines. The potential pathogenic mechanisms were explored by the analysis of the interactive partners of beta-actin and the impact of the identified variants in molecular docking. The underlying mechanism of phenotypic features was investigated by the spatiotemporal expression of *ACTB*. The present study suggested the *ACTB* gene as one of the novel candidate causative genes of TLE. The correlation between phenotypes and spatiotemporal expression provides a novel perspective for further exploration of the pathogenesis and prognosis of the disease.

## 2. Materials and Methods

### 2.1. Subjects

The patient was recruited from Guangdong Sanjiu Brain Hospital. Epileptic seizures and epilepsy syndromes were diagnosed according to the criteria of the Commission on Classification and Terminology of the International League Against Epilepsy (2022). TLE was diagnosed according to the following features: (1) focal original seizures or focal seizures manifesting automatic symptoms, mental symptoms, special sensory symptoms such as vision and auditory hallucinations, and/or gastrointestinal symptoms and (2) interictal focal dischargers in the temporal lobe detected by electroencephalogram (EEG).

This study received approval from the Ethics Committee of the Guangdong Sanjiu Brain Hospital. Written informed consents were provided by the patient's legal guardians.

### 2.2. WES

Genomic DNAs were extracted from blood samples of the probands and the parents to perform WES by a NextSeq500 sequencing instrument (Illumina, San Diego, California, United States) following the standard procedures previously described [11–14]. High-quality sequencing data were acquired through massively parallel sequencing, achieving an average depth of over 125× and more than 98% coverage of the capture region on the chip. These reads were then successfully mapped to the Genome Reference Consortium Human Build 37 using Burrows–Wheeler alignment. The Genome Analysis Toolkit was employed to identify single-nucleotide point variants and indels.

### 2.3. Damaging Effect Analysis

The consequences of all the missense variants were predicted by 18 common in silico tools, including PolyPhen2_HVAR [15], LRT [16], MutationTaster [17], FATHMM [18], PROVEAN [19], CADD [20], MetaSVM [21], MetaLR, M_CAP [22], FATHMM_MKL [23], Eigen [24], GenoCanyon [25], fitCons [26], GERP [27], phyloP [28], phastCons [29], REVEL [30], and ReVe [31]. Protein modeling was performed by using the three web tools, including the SWISS model software (April 2024 version, https://swissmodel.expasy.org/), the Phyre2 model (http://www.sbg.bio.ic.ac.uk/phyre2/html/page.cgi?id=index), and the AlphaFold model (monomer v2.0, https://alphafold.com/). PyMOL Molecular Graphics System (version 2.3.2; Schrödinger, LLC; New York, United States) was used for three-dimensional protein structure visualization and analysis. Molecular docking was conducted by the ZDOCK Server (https://zdock.umassmed.edu/). The hydrophobicity changes of variants were analyzed by the VarSite web server [32]. The pathogenicity of variants was evaluated by the ACMG guideline [33].

### 2.4. Assessment of *ACTB* Expression Profile

The expression profile of *ACTB* was assessed by the method of our previous studies [34–36]. Expression in adult 54 nondisease tissues was analyzed by the data from the Genotype-Tissue Expression (GTEx) database (https://www.gtexportal.org/home/). Human RNA-seq data of developmental stages (from eight postconceptional weeks to 40 years) for multiple brain areas were obtained from the BrainSpan database (http://www.brainspan.org/). RNA expression was normalized to reads per kilobase million (RPKM). The expression spline is fitted by the locally weighted scatterplot smoothing (LOWESS) algorithm for interpreting the expression pattern of *ACTB*.

### 2.5. Protein–Protein Interaction (PPI) Network Analysis

The PPI network of the ACTB protein was analyzed using the STRING database (https://string-db.org/; version: 12.0) [37, 38]. The interactive genes with a confidence score ≥ 0.9 were taken into analysis, with basic settings including (1) “physical subnetwork” for network type, (2) “evidence” for the meaning of network edges, (3) “experiments” and “databases” for active interaction sources, and (4) “500 max interactors (max)” for max number of interactors to show.

### 2.6. Statistical Analysis

R (version 4.0.3) was used for data processing. *p* value and/or *Q*‑value (false discovery rate) < 0.05 was considered statistically significant.

## 3. Results

### 3.1. Identification of *ACTB* Variant and Clinical Feature

**Figure 1:**
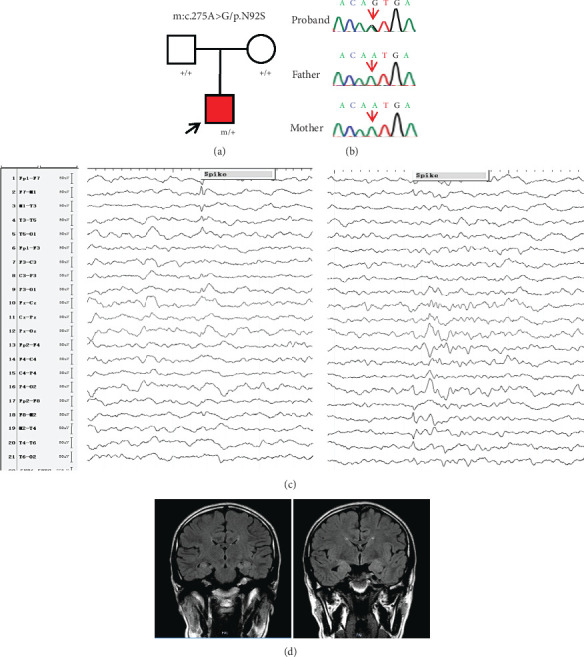
Genetic data and representative clinical images. (a) Pedigrees of the case with *ACTB* variant. (b) DNA sequencing chromatograms of the case with *ACTB* variant. Red arrows indicate the positions of the variant. (c) Representative EEG showed focal discharges in the bilateral hippocampi. (d) Representative MRI indicated bilateral hippocampal sclerosis.

In this study, one variant in the *ACTB* gene (c.275A>G/p.N92S) was identified in a patient (Figures 1(a) and 1(b)). The variant was of de novo and absent in any controls, consistent with the Mendelian-dominated inherited pattern (Table 1).

The patient was born to nonconsanguineous parents, was delivered normally, experienced neurodevelopment consistent with peers, and had no facial dysmorphism. When the patient was 17 years old, the first seizure occurred, manifesting focal features including nausea, headaches, and subsequent loss of consciousness. The seizures were repeatedly exhibited two or three times per year. Two years later, the patient exhibited another type of seizure, focal original tonic–clonic seizures, manifesting loss of consciousness, upward rolling of the eyes, and convulsions of the limbs during sleep. Then, he was referred to our hospital and received the treatment of levetiracetam tablets (1 g/day). After 3 months of treatment with levetiracetam, seizures still attacked. Another antiseizure medicine, lamotrigine (50 mg/day), was thus added. Subsequently, the patient achieved a seizure-free status for more than 2 years, until now. The patient also reported a decline in memory and some irritability since the first seizure, which has improved slightly recently. Focal discharges were detected in the bilateral temporal lobes (Figure 1(c)) by EEG before being treated with lamotrigine, but normal in the past 2 years. Magnetic resonance imaging scans detected bilateral hippocampal sclerosis (Figure 1(d)). Taken together, the clinical features and epileptic discharges, the patient was diagnosed with TLE.

### 3.2. The Pathogenicity of the Identified *ACTB* Variant

**Figure 2:**
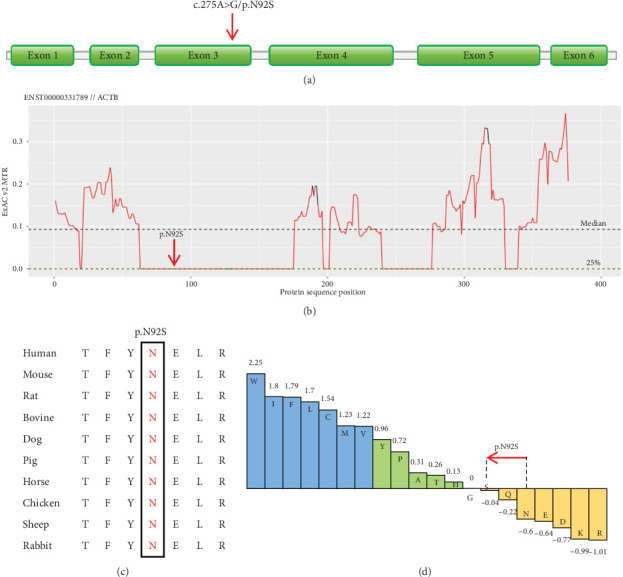
Location and molecular effects of the identified variant. (a) Location of the variant c.275A>G/p.N92S in the *ACTB* gene. (b) Landscape of missense tolerance ratio for the *ACTB*. Data was obtained from the MTR Gene Viewer. (c) Amino acid sequence alignment of the residue of Asn-92 across species. (d) Hydrophobicity plot, in order of decreasing hydrophobicity.

The identified variant, c.275A>G/p.N92S, was located in the third exon of the *ACTB* gene (Figure 2(a)). According to the Missense Tolerance Ratio (MTR) Gene Viewer developed to assess the pathogenicity of missense variants [39], the variant affects a residue of a missense tolerance ratio of 0% (Figure 2(b)). Cross-species sequence alignment suggests that this variant occurs in a residue with high conservation (Figure 2(c)). The variant is also predicted to decrease the hydrophobicity, calculated by the Fauchère and Pliska hydrophobicity scale (Figure 2(d)). Notably, the variant is predicted to be “damaging” or “conserved” by almost all in silico tools (17/18 tools, Table 2). According to the guidelines of the ACMG, this variant is classified as “likely pathogenic,” due to the origination of de novo (PS2), not presented in populations (PM2), and predicted to be damaging by multiple in silico tools (PP3).

Additionally, no “likely pathogenic” or “pathogenic” variants in other genes are identified in this case, according to the ACMG guidelines (Table S1). The identified *ACTB* variant is thus considered to be the genetic explanation of this case.

### 3.3. The Interacted Protein of Beta-Actin and Their Associated Function, Pathways, and Phenotypes

**Figure 3:**
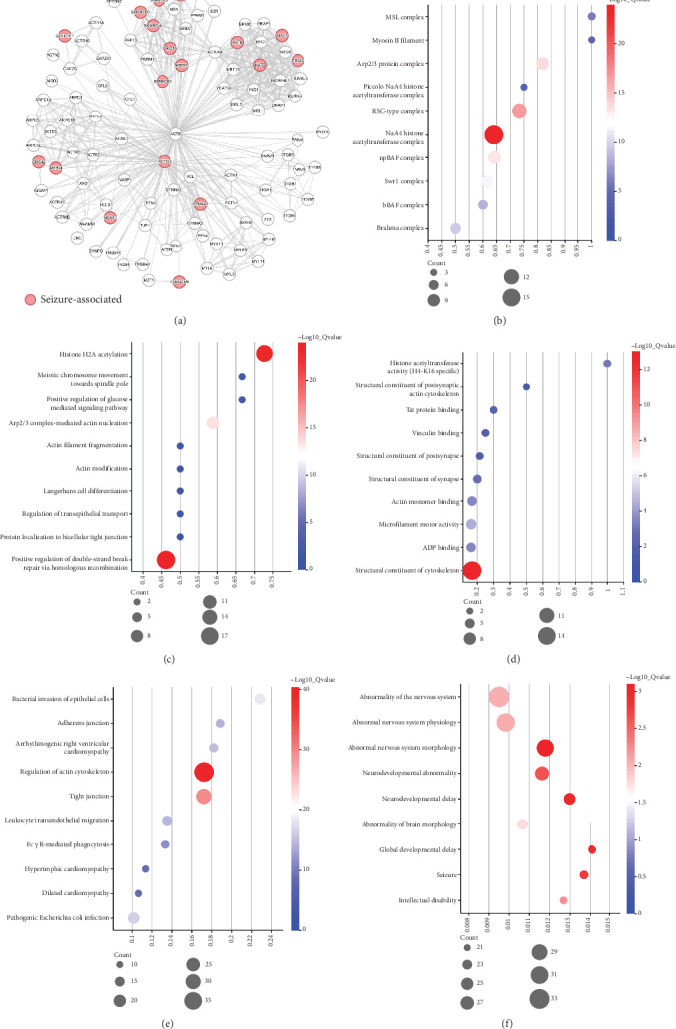
Protein–protein interaction network and its enrichment analysis of *ACTB.* (a) Protein–protein interaction network produced by the STRING v12. (b–d) Each top 10 GO enrichment analysis of all node genes in cellular components (b), biological processes (c), and molecular functions (d), ranked by log10 (observed/expected). (e) Top 10 KEGG enrichment analysis of all node genes. (f) HPO term enrichment analysis of all node genes; nervous system phenotypes of top 10 are shown.

The *ACTB* gene encodes beta-actin protein, a highly conserved protein that polymerizes to produce filaments that form cross-linked networks that are ubiquitous in the cytoplasm of diverse cells. Actin exists in both monomeric (G-actin) and polymeric (F-actin) forms, both forms interact with numerous proteins, playing key functions, such as cell motility and contraction. In addition to their role in the cytoplasmic cytoskeleton, G- and F-actin also localize in the nucleus and regulate gene transcription and motility and repair of damaged DNA. To explore the underlying pathogenic mechanism, we investigated the PPI network of the beta-actin protein via the STRING database. The PPI network of *ACTB* included 100 nodes, 530 edges, 10.6 of average node degree, 0.907 of average local clustering coefficient, and the PPI enrichment *p* value < 1.0 × 10^−16^ (Figure 3(a)). The PPI included numerous genes associated with seizures, such as *ARID2*, *ARID1A*, *CDC42*, *CTNNA2*, *DYNC1H1*, *TRRAP*, and *SMARCA4* (Online Mendelian Inheritance in Man (OMIM) database, https://omim.org/).

Meanwhile, lots of neurodevelopmental-related terms were significantly enriched on genes of the PPI network. The top 10 cellular components of Gene Ontology (GO) terms showed that these genes are involved in chromatin structure regulation (male-specific lethal (MSL) complex, NuA4 histone acetyltransferase complex, Swr1 complex, etc.) or participate in cell movement and morphogenesis (myosin II filament, arp2/3 protein complex, etc.), which contribute to proper neuronal differentiation and synapse formation (Figure 3(b)) [40, 41]. The top 10 biological processes of GO terms showed similar localization of function in the above components, namely, chromatin remodeling or actin cytoskeleton activity (Figure 3(c)). The top 10 molecular functions of GO terms showed that 30% of pathways are directly related to synapses, and 60% indirectly affect the formation of synapses or other neuronal structures (Figure 3(d)). The top 10 Kyoto Encyclopedia of Genes and Genomes (KEGG) terms showed that these node genes are involved in the regulation of actin cytoskeleton, which may be underlying pathogenic pathways of actin causing epilepsy and/or neurodevelopmental disorders (Figure 3(e)) [42, 43]. Similarly, phenotypic enrichment analysis of these genes from the Monarch database revealed several significantly enriched Human Phenotype Ontology (HPO) terms associated with various abnormalities of the nervous system, including seizure (Figure 3(f)). The enrichment analysis revealed the vital roles of *ACTB* in epilepsy/neurodevelopment. Variants in the *ACTB* gene may disrupt its interaction with these genes to cause the phenotypes of neurodevelopmental disorders and epilepsy.

### 3.4. Protein Modeling and Molecular Docking

**Figure 4:**
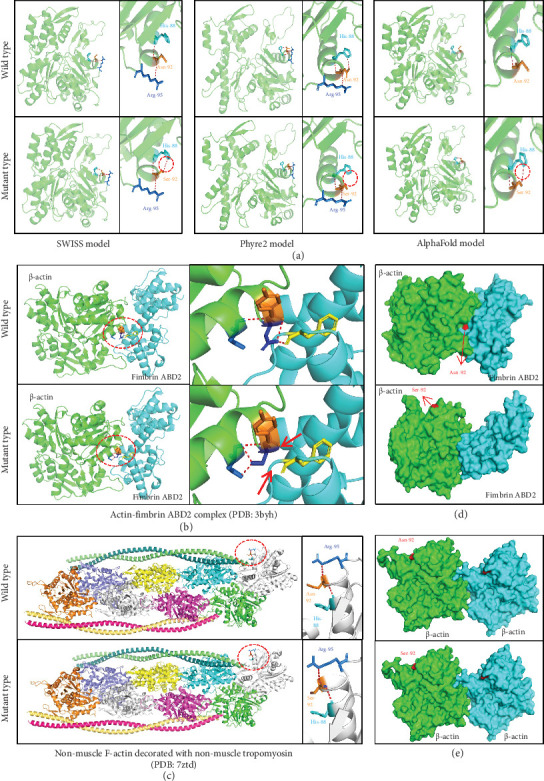
Protein modeling and molecular docking of the identified variant. (a) Protein modeling via three advanced artificial intelligence algorithms: the SWISS model, the Phyre2 model, and the AlphaFold model. (b) The molecular structural alteration of the variant of the actin-fimbrin ABD2 complex (PDB: 3byh). (c) The molecular structural alteration of the variant of the nonmuscle F-actin decorated with nonmuscle tropomyosin complex (PDB: 7ztd). (d) The alteration of the variant in molecular docking between beta-actin and fimbrin ABD2. (e) No alteration of the variant in molecular docking between beta-actin and nonmuscle tropomyosin.

Given the crucial role of beta-actin as a scaffolding protein, we evaluated the structural implications of the identified variant c.275A>G/p.N92S. This assessment was conducted by protein modeling, through three advanced artificial intelligence algorithms: the SWISS model, the Phyre2 model, and the AlphaFold model. Notably, the substitution of asparagine with serine at Residue 92 resulted in significant alterations to the hydrogen bonding patterns, calculated by all three models. Specifically, in all three models, we observed altered hydrogen bonds previously established between Residue 92 and the histidine residues at Position 88 (Figure 4(a)).

The consequence of the variant on the monomeric (G-actin) and polymeric (F-actin) forms of beta-actin was further investigated, by two representative structures of experimental verification. One of the structures is the actin-fimbrin actin-binding domain 2 (ABD2) complex (Protein Data Bank (PDB): 3byh) [44], formed by a beta-actin and a fimbrin ABD2 and representing the monomeric (G-actin) forms. Another structure is the nonmuscle F-actin decorated with nonmuscle tropomyosin complex (PDB: 7ztd) [45], formed by eight beta-actin and four nonmuscle tropomyosin 3.2 and representing the polymeric (F-actin) forms. This variant was predicted to alter the hydrogen bonds that were previously linked to the residue of fimbrin ABD2 (Figure 4(b)) but showed less impact on the formation of polymeric beta-actin (Figure 4(c)), indicating the damaging effects of molecular docking with other proteins but not self-polymerization. We thus investigated the impact of this variant in molecular docking. It is shown that the variant altered molecular docking between beta-actin and fimbrin ABD2 (Figure 4(d)), but not molecular docking of self-polymerization (Figure 4(e)). This finding underscores the impact of this variant on the structural integrity of beta-actin and its potential functional consequences, which may be evolved in the pathogenesis.

### 3.5. The Spatial–Temporal Expression of ACTB

**Figure 5:**
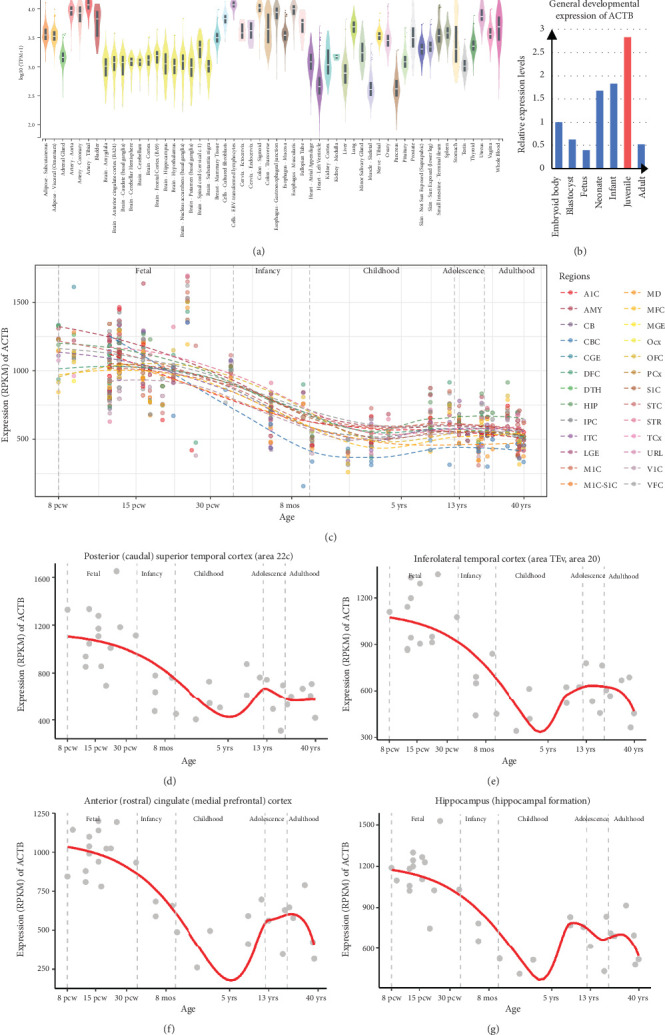
The spatiotemporal expression of the *ACTB* gene. (a) RNA expression of *ACTB* in normal adult tissues, shown by the data from the GTEx database (http://www.gtexportal.org/home/gene/actb). (b) General developmental expression of *ACTB*, presented with the data from the NCBI-UniGene database (http://www.gdap.org.cn/). (c–g) Developmental expression of *ACTB* in varied brain regions. Data were obtained from the BrainSpan database. The temporal expression curve was fitted by the Lasso algorithm. Abbreviations: A1C: primary auditory cortex; AMY: amygdaloid complex; CB: cerebellum; CBC: cerebellar cortex; CGE: caudal ganglionic eminence; DFC: dorsolateral prefrontal cortex; DTH: dorsal thalamus; HIP: hippocampus; IPC: posteroventral parietal cortex; ITC: inferolateral temporal cortex; LGE: lateral ganglionic eminence; MD: mediodorsal nucleus of thalamus; MFC: anterior cingulate cortex; MGE: medial ganglionic eminence; M1C: primary motor cortex; M1C-S1C: primary motor–sensory cortex; OFC: orbital frontal cortex; Ocx: occipital neocortex; PCx: parietal neocortex; S1C: primary somatosensory cortex; STR: striatum; STC: posterior superior temporal cortex; TCx: temporal neocortex; URL: upper rhombic lip; V1C: primary visual cortex; VFC: ventrolateral prefrontal cortex.

TLE is generally intractable epilepsy with onset age mostly in adults [1], while the patient of this study had the age of onset in juvenile and achieved seizure-free status in adulthood. The spatiotemporal expression stands as one of the pivotal characteristics of genes, intricately linked to the phenotypes they cause. The spatiotemporal expression of *ACTB* was thus investigated. The *ACTB* gene is ubiquitously expressed in multiple adult tissues, including the brain cortex, temporal lobe, hippocampus, and other brain regions, showed by the data obtained from the GTEx database (Figure 5(a)). *ACTB* showed fluctuating expression at different development stages, exhibiting the highest expression in juveniles, according to the NCBI-UniGene database (Figure 5(b)). According to the specific brain expression database BrainSpan, in most brain regions, the expression of *ACTB* is highest in fetal but decreased after birth with minimum expression at approximately 4 years old, exhibiting a development-dependent feature (Figure 5(c)). A slightly increased expression of *ACTB* was observed in juveniles. Further investigation of regions associated with the phenotypes of this study showed that the second peak of *ACTB* expression in the temporal lobe and anterior cingulate cortex was in the stage of juvenile but decreased in adulthood (Figures 5(d), 5(e), and 5(f)), consisting of the phenotypic feature of this patient. The second peak of *ACTB* expression in the hippocampus is in the stage of later childhood (Figure 5(g)), which may be the explanation for bilateral hippocampal sclerosis detected in juveniles. Overall, the expression of *ACTB* consisted of the clinical and imaging features of this patient.

## 4. Discussion

In this study, we identified a de novo *ACTB* variant in a patient. The patient presented with TLE with clinical features including the age of onset in juvenile, focal or focal original seizures with occasional precursors of nausea and/or headaches, seizure-free status in adulthood, complications of memory decline and irritability, epileptic discharges in the bilateral temporal lobes, and bilateral hippocampal sclerosis. The pathogenicity of the identified *ACTB* variant was supposed by multiple pieces of evidence, including the missense tolerance ratio of 0% calculated by MTR Gene Viewer, the affected residue of high conservation, hydrophobicity alteration, predicted to be “damaging” or “conserved” by almost all in silico tools, and classification of likely pathogenic variant by the ACMG/Association for Molecular Pathology (AMP) guidelines. Protein modeling indicated the alteration of protein structure and molecular docking by the identified variant. Investigation of spatial–temporal expression indicated a consistent pattern of *ACTB* expression with phenotypic features of this patient. This study suggested that *ACTB* is a novel candidate causative gene of TLE.

Epilepsy is one of the most common neurological disorders, accounting for more than 0.5% of the global burden of disease, according to the World Health Organization (WHO). Patients with epilepsy may be caused by acquired factors, such as infectious, metabolic, immune, and tumor [46]. However, the cause in most epileptic cases remains undetermined. Genetic factors are generally believed to be the major causes of epilepsy, which are estimated to contribute to 80% of people with epilepsy [47, 48]. Over the last two decades, 168 core epilepsy genes have been identified, among which 115 genes (68.5%) were linked to the severe and rare epilepsy of developmental and epileptic encephalopathy [49]. Only four genes were reported to be possibly associated with the common epilepsy of TLE, including *LGI1*, *CPA6*, *GAL*, and *RELN*. This study identified *ACTB* as a novel candidate causative gene of TLE, providing new targets for future precision medicine.

In genetics, the expression pattern of a gene often correlates closely with the phenotypic features exhibited in individuals. Our study found that *ACTB* is highly expressed before birth and during juveniles, which provides important clues to our understanding of the role of this gene in the development of disease. It is particularly noteworthy that the patient in this study mainly presented with TLE with age of onset in juveniles and seizure-free status in adulthood, which is consistent with the high expression pattern of *ACTB* in juveniles and decreased expression in adulthood. The association between *ACTB* and TLE was further supported by the spatial–temporal expression-phenotype correlation, which also provides a new perspective for further exploration of the pathogenesis and prognosis of the disease.

Based on the theory of natural selection, sequencing data of large-cohort populations have calculated gene-level constraint metrics for the *ACTB* gene, including 0.19 for the loss-of-function observed/expected upper bound fraction (LOEUF) index, 1 for the probability of being loss-of-function intolerant (pLI) index, and 7.69 of the missense *Z*-score [50]. The three constraint indexes suggest that the observed amount of loss-of-function and missense variants of the *ACTB* gene was significantly lower than the expected amount, emphasizing the high intolerance of both loss-of-function and missense variants for the *ACTB* gene. The identified variant in this study, c.275A>G/p.N92S, was located in the region with the missense tolerance ratio of 0%, predicted to be damaging by multiple in silico tools, and classified to be “likely pathogenic” by the ACMG/AMP guidelines. Additionally, no “likely pathogenic” or “pathogenic” variants in other genes are identified in this case. This variant is thus considered to be the diseasing-causing variant for this case.

The *ACTB* gene encodes the beta-actin protein which is an integral component of cellular structure and function. The beta-actin protein, via monomeric and polymeric forms, interacts with numerous proteins to play critical roles in a wide range of cellular processes, such as cytoskeleton maintenance, cell motility, and cell signal transduction. The PPI network analysis indicated that proteins that interacted with beta-actin protein were widely associated with function, pathways, and phenotypes associated with neurodevelopment/epilepsy. The identified variant, c.275A>G/p.N92S, caused not only structural alterations in the hydrogen bonds that were previously established with the Histidine Residue 88 but also abnormal molecular docking, which was subsequently speculated to alter the inaction of other neurodevelopmental and epilepsy-related protein to be involved in the occurrence of TLE. However, the detailed functional alteration of this variant needed to be experimentally characterized.

Despite the significant insights gained from this study on the association between the *ACTB* gene and TLE, a limitation needs to be acknowledged. Only one case was included in this study, due to the high constraint of variants for *ACTB*. The phenotypes of TLE and the whole phenotypic spectrum of *ACTB* warrant large cohort studies.

In summary, this study has revealed *ACTB* as a novel candidate causative gene of TLE. The spatial–temporal expression pattern of genes helps in understanding the association between genetic basis and phenotype, providing new insight for future precision medicine.

## Figures and Tables

**Table 1:** Minor allele frequency of the identified *ACTB* variant c.275A>G/p.N92S.

**Population**	**Allele count**	**Allele numbers**	**Allele frequency**
gnomAD (V4.0.0)^a^	0	1,614,324	0
Epi25 WES Browser^b^	0	108,846	0
PGG.Han (2.0)^c^	0	274,024	0

^a^Data from the gnomAD database (https://gnomad.broadinstitute.org/).

^b^Data from Epi25 WES Browser (https://epi25.broadinstitute.org/).

^c^Data from PGG.Han 2.0 databases (https://www.biosino.org/pgghan2/index).

**Table 2:** Prediction of damaging effects by 18 in silico tools.

**Algorithm**	**Score**	**Prediction**
Polyphen-2_HVAR	0.544	Possibly damaging
LRT	0.000	Deleterious
MutationTaster	0.945	Disease causing
FATHMM	−3.4	Damaging
PROVEAN	−3.54	Damaging
CADD	16.02	Tolerable
MetaSVM	0.988	Damaging
MetaLR	0.866	Damaging
M-CAP	0.454	Damaging
FATHMM_MKL	0.979	Damaging
Eigen	0.439	Damaging
GenoCanyon	1.000	Damaging
fitCons	0.752	Damaging
GERP	3.83	Conserved
phyloP	6.190	Conserved
phastCons	1.000	Conserved
REVEL	0.614	Damaging
ReVe	0.963	Pathogenic

Abbreviations: CADD, combined annotation dependent depletion; FATHMM, functional analysis through hidden Markov models; fitCons, the fitness consequences of functional annotation; GERP, genomic evolutionary rate profiling; LRT, likelihood ratio test; M-CAP, Mendelian clinically applicable pathogenicity score; phastCons, conservation scoring and identification of conserved elements; phyloP, computation of *p* values for conservation or acceleration, either lineage-specific or across all branches; Polyphen-2_HVAR, HumVar-trained PolyPhen-2; PROVEAN, protein variation effect analyzer; ReVe, a combination of the predictions of REVEL and VEST4 (variant effect scoring tool 4.0); REVEL, rare exome variant ensemble learner.

## Data Availability

Data are available from the corresponding author upon reasonable request.

## Supporting Information

Supporting Information Additional supporting information can be found online in the Supporting Information section. Table S1 Mendelian variants identified by WES in the case and associated genetic characteristics.
