# Supplementary material for: De Novo ACTB Variant Associated With Juvenile-Onset Temporal Lobe Epilepsy With Favorable Outcomes
Source: Hum Mutat. 2025 Feb 12;2025:9951922. doi: 10.1155/humu/9951922 (PMC12267973; doi:10.1155/humu/9951922)
Supplement: Supporting Information — Additional supporting information can be found online in the Supporting Information section. Table S1 Mendelian variants identified by WES in the case and associated genetic characteristics. [file 9951922.f1.docx]

**Table S1. Mendelian variants identified by WES in the case and associated genetic characteristics.**

| **Gene** | **ACTB** | **ALDH16A1** | | **L1CAM** | **FLNA** |
| --- | --- | --- | --- | --- | --- |
| **Chr** | chr 7 | chr 19 | chr 19 | chr X | chr X |
| **Position** | 5568880 | 49973630 | 49963010 | 153133485 | 153592449 |
| **Nucleotide change** | c.275A>G | c.2315C>T | c.404G>A | c.1796T>C | c.2221G>A |
| **Amino acid change** | p.N92S | p.P772L | p.R135Q | p.V599A | p.V741M |
| **Inheritance** | De novo | Paternal | Maternal | Maternal | Maternal |
| **MAF*** | - | 0.00001736 | 0.0001177 | 3.305E-06 | 3.307E-06 |
| **Homozygous/hemizygous number*** | - | 0 | 0 | 0 | 1 |
| **OMIM (Updated July 12th, 2024)** | Baraitser-Winter syndrome 1, AD [BRWS1 (MIM: 243310)]; Dystonia-deafness syndrome 1, AD [DDS1 (MIM: 607371)]; Thrombocytopenia 8, with dysmorphic features and developmental delay, AD [THC8 (MIM: 620475)]. | - | | ?Corpus callosum, partial agenesis of, XLR [CCPAX (MIM: 304100)]; Hydrocephalus, congenital, X-linked, XLR [HYCX (MIM:307000)]; MASA syndrome, XLR [MASAS/SPG1 (MIM:303350)]. | ?FG syndrome 2  XL [FGS2 (MIM: 300321)]; Cardiac valvular dysplasia, X-linked XL [CVDPX (MIM: 314400)]; Congenital short bowel syndrome, XLR [CHRONIC/CIIPX (MIM: 300048)]; Frontometaphyseal dysplasia 1, XLR [FMD (MIM: 305620)]; Heterotopia, periventricular, 1, XLD [PVNH1 (MIM: 300049)]; Intestinal pseudoobstruction, neuronal, XLR [CHRONIC/CIIPX (MIM: 300048)];  Melnick-Needles syndrome, XLD [MNS (MIM: 309350)]; Otopalatodigital syndrome, type I, XLD [OPD1 (MIM: 311300)]; Otopalatodigital syndrome, type II, XLD [OPD2 (MIM: 304120)]; Terminal osseous dysplasia, XLD [TOD (MIM: 300244)]. |
| **Knockout (Updated July 2nd, 2024, MGI database)** | Homozygous null mutants are embryonic lethal; Homozygotes for a hypomorphic targeted mutation develop normally until embryonic day 8.5 are growth retarded by day 9.5 and die shortly thereafter. (MGI: 87904) | Homozygous knockout mice exhibit abnormal blood plasma lipid and ion profiles. (MGI: 1916998) | | Homozygous null mutants have reduced size, lessened sensitivity to touch and pain, weakness and incoordination of hind-legs, reduced corticospinal tract, impaired guidance of retinal and corticospinal axons, and in some cases, enlarged lateral ventricles. A hypomorphic line shows background effects. (MGI: 96721) | Females heterozygous for an X-linked, ENU-induced mutation exhibit dilated pupils and milder cardiac, sternum, and palate defects than males. Hemizygous males are inviable and exhibit incomplete septation of the outflow tract, septal defects, cleft palate and incomplete fusion of the sternum. (MGI: 95556) |
| **Polyphen-2_HVAR** | PD(0.544) | PD(0.591) | PD(0.998) | B(0.019) | PD(0.48) |
| **LRT** | D(0.000) | N(0.783) | N(0.188) | N(0.757) | D(0.000) |
| **MutationTaster** | PD(0.945) | Po(1) | D(1) | Po(1) | D(0.985) |
| **FATHMM** | D(-3.4) | T(-0.47) | T(-1.11) | T(-0.16) | T(-0.61) |
| **PROVEAN** | D(-3.54) | T(-0.87) | D(-3.45) | T(1.09) | T(-1.52) |
| **CADD** | T(16.02) | T(0.224) | D(27.5) | T(0.070) | D(22.9) |
| **MetaSVM** | D(0.988) | T(-0.896) | D(0.250) | T(-1.006) | T(-0.399) |
| **MetaLR** | D(0.866) | T(0.181) | D(0.614) | T(0.033) | T(0.335) |
| **M-CAP** | D(0.454) | D(0.045) | D(0.095) | D(0.030) | D(0.029) |
| **FATHMM_MKL** | D(0.979) | T(0.011) | D(0.891) | T(-0.16) | T(0.102) |
| **Eigen** | D(0.439) | T(-1.706) | D(0.546) | - | - |
| **GenoCanyon** | D(1.000) | D(1.000) | D(1.000) | T(0.098) | D(1.000) |
| **fitCons** | D(0.752) | D(0.731) | D(0.713) | - | - |
| **GERP+++** | C(3.83) | NC(-2.97) | C(5.38) | NC(0.403) | C(4.95) |
| **phyloP** | C(6.190) | NC(-0.993) | C(5.086) | NC(-1.168) | NC(0.859) |
| **phastCons** | C(1.000) | NC(0.000) | NC(0.999) | NC(0.000) | NC(0.289) |
| **REVEL** | D(0.614) | T(0.050) | D(0.628) | T(0.036) | T(0.269) |
| **ReVe** | D(0.96274518) | B(0.01071779) | B(0.06119800) | B(0.01439254) | B(0.16522927) |
| **ACMG** | PS2+PM2+PP3(LP) | PM2(US) | PM2(US) | PM2(US) | PM2(US) |
| **Comments** | 1) Classified as likely pathogenic by ACMG guidelines | 1) Normal neurological phenotype in KO mice;  2) Unknown gene-disease association; 3) Predicted to be "Benign" by majority of in silico tools; 4) Classified as uncertain significance by ACMG guidelines | | 1) Normal neurological phenotype in KO mice;  2) Predicted to be "Benign" by majority of in silico tools; 3) Classified as uncertain significance by ACMG guidelines | 1) Normal neurological phenotype in KO mice;  2) Predicted to be "Benign" by majority of in silico tools; 3) Classified as uncertain significance by ACMG guidelines |

The Mendelian variants were included, according to the following criteria: (1) *De novo* variants: not presented in the controls of gnomAD; (2) hemizygous variants: no hemizygote in controls of gnomAD; (3) homozygous variants: no homozygote in the controls of gnomAD; (4) compound heterozygous variants: the frequency of a single variant of a pair of compound heterozygous variants < 0.005 in gnomAD-all population.

*Only the frequency of variants in gnomAD-all populations (gnomAD v4.1.0) was provided.

Abbreviations: AD, autosomal dominant; AR, autosomal recessive; B, benign; C, conserved; CADD, Combined Annotation Dependent Depletion; D, damaging; FATHMM-MKL, Functional Analysis through Hidden Markov Models–Multiple Kernels Learning; fitCons, the fitness consequences of functional annotation; GERP++, Genomic Evolutionary Rate Profiling; KO: knock-out mouse model; LRT, likelihood ratio test; M_CAP, Mendelian Clinically Applicable Pathogenicity; MAF, minor allele frequency in gnomAD-all populations; NC, Nonconserved; OMIM, Online Mendelian Inheritance in Man ; P, pathogenic; Po, polymorphism; PD, probably damaging; phastCons, Phylogenetic Analysis with Space/Time models conservation scoring and identification of conserved elements; phyloP, Phylogenetic Analysis with Space/Time models Computation of p-values for conservation or acceleration, either lineage-specific or across all branches; pLi,probability of being loss-of-function intolerant (both heterozygous and homozygous lof variants); Polyphen-2_HVAR, HumVar-trained PolyPhen-2; PROVEAN, protein variation effect analyzer; REVEL, rare exome variant ensemble learner; ReVe, a combination of the predictions of REVEL and VEST4 (variant effect scoring tool 4.0), T, tolerable; WES, whole exome sequencing; XL, X-linked; XLD, X-linked dominant; XLR, X-linked recessive.
